# A new species of *Mongolodiaptomus* Kiefer, 1938 from northeast Thailand and a key to the species (Crustacea, Copepoda, Calanoida, Diaptomidae)

**DOI:** 10.3897/zookeys.710.13941

**Published:** 2017-10-19

**Authors:** Santi Watiroyram, La-orsri Sanoamuang

**Affiliations:** 1 Division of Biology, Faculty of Science, Nakhon Phanom University, Nakhon Phanom 48000, Thailand; 2 Applied Taxonomic Research Center, Khon Kaen University, Khon Kaen 40002, Thailand; 3 International College, Khon Kaen University, Khon Kaen 40002, Thailand

**Keywords:** Copepoda, Diaptomidae, Loei Province, new record, rare species, southeast Asia, taxonomy

## Abstract

This study describes the new species *Mongolodiaptomus loeiensis* **sp. n.** collected from a temporary pond nearby a cave located in Loei Province, in northeastern Thailand. *Mongolodiaptomus loeiensis* **sp. n.** is similar to *M. calcarus* (Shen & Tai, 1965) in the male but can be distinguished from its congeners by the following unique characteristics in the males: (1) the right caudal ramus has 3 ventral chitinous prominences; (2) intercoxal plate of P5 is produced into 2 spine-like lobes on distal margin; (3) the basis of right P5 has a subglobular chitinous prominence on mid-distal caudal surface; and (4) the principal lateral spine on the right Exp-2 P5 is extremely bent at its tip. The occurrence of diaptomid copepods in the study area is discussed and an identification key to worldwide species of the genus *Mongolodiaptomus* Kiefer, 1938 is presented herein.

## Introduction

The freshwater calanoid copepods have been intensively studied in Thailand especially in northeast Thailand. However, their study in the Loei Province in the upper northeast region has so far been largely neglected. According to Sanoamuang (2002), only three diaptomid species were previously recorded in Loei Province, namely *Mongolodiaptomus botulifer* (Kiefer, 1974), *M. calcarus* (Shen & Tai, 1965), and *Phyllodiaptomus praedictus* (Dumont & Reddy, 1994). During the years 2014–2016, the first author had led sampling surveys on planktonic and cave-dwelling copepods in the upper northeastern region in order to fill the gap of copepod richness and distribution in this region (Watiroyram et al. 2015, 2017). As results of this study, *Mongolodiaptomus loeiensis* sp. n. and other diaptomids were discovered in water bodies outside the caves.

The genus *Mongolodiaptomus* Kiefer, 1938 was defined by Kiefer (1938) by several characters, especially in the male fifth leg. Nevertheless, some of these characters are not useful for separating the species because they are shared by certain species of *Allodiaptomus* Kiefer, 1936 and *Neodiaptomus* Kiefer, 1932. After the revision of Reddy et al. (2000), the solution on diagnosis of problematic species was well defined on generic characters. Based on this revision, *Mongolodiaptomus* is characterized by the second exopod of the right male P5 having 3 spines and processes on its outer margin; one principal spine somewhat on middle of segment, and 1–2 spinous processes proximally or/and distally. As a result, nine species of *Allodiaptomus, Diaptomus* and *Neodiaptomus* were transferred into the genus *Mongolodiaptomus*; namely *M. birulai* (Rylov, 1922), *M. botulifer*, *M. calcarus*, *M. gladiolus* (Shen & Lee, 1963), *M. malaindosinensis* (Lai & Fernando, 1978), *M. mephistopheles* (Brehm, 1933), *M. pectinidactylus* (Shen & Tai, 1964), *M. rarus* (Reddy, Sanoamuang & Dumont, 1998), *M. uenoi* (Kikuchi, 1936) (for more details see Kiefer 1939; Reddy et al. 1998, 2000; Sanoamuang 1999, 2002; Luong et al. 2016).

To date, 37 diaptomid species are known from inland waters of Thailand (Sanoamuang 2002). Of these, seven species belong to the genus *Mongolodiaptomus*: *M. malaindosinensis*, *M. botulifer*, *M. calcarus*, *M. dumonti* Sanoamuang, 2001, *M. pectinidactylus*, *M. rarus*, and *M. uenoi*. *Mongolodiaptomus loeiensis* sp. n., the eighth species from Thailand, is illustrated and described herein together with a dichotomous key to the worldwide species of the genus *Mongolodiaptomus*. Additionally, the geographical distribution of the recorded diaptomids in Loei Province is briefly discussed.

## Material and methods

Samples were collected using a plankton net with a mesh size of 60 µm. The copepod samples were transferred into 120 ml plastic bottles and preserved in 70% ethanol. In the laboratory, samples were selected for individual adults and placed in a mixture of glycerol and 70% ethanol (ratio ~ 1:10 v/v) under a stereomicroscope at 40× magnification. Specimens were transferred to pure glycerol and dissected at 40–100-× magnification under an Olympus SZ51 stereomicroscope.

All appendages and body ornamentation were examined at1000-× magnification. All the drawings were made at the same magnification (1000-×), with a drawing tube mounted on an Olympus compound microscope (CX31). The final versions of the drawings were made using the CORELDRAW^®^ 12.0 graphic program. For permanent slides, all body parts were put in a drop of glycerol on a microscope slide, covered by a cover glass, and sealed with nail polish.

Specimens for a scanning electron microscopy (SEM) were dehydrated in an ethanol series (50%, 70%, 80%, 90%, 95%, 100%, and 100%) for 15 min each concentration. After dehydration, specimens were dried in a critical point dryer using liquid carbon dioxide as the exchange medium. Dried specimens were mounted on stubs using adhesive tape under a stereomicroscope. Then, specimens were coated with gold in a sputter-coater. The SEM photographs were carried out using a scanning electron microscope (FEI Helios NanoLab G3 CX).

### The following abbreviations are used throughout the text and figures

**Enp** endopod;

**Exp** exopod;

**Exp/Enp-n** exopodal segment n/endopodal segment n;

**P1–P5** swimming legs 1–5.

The nomenclature and descriptive terminology follow Huys and Boxshall (1991), including analysis of caudal setae (I–VII). Specimens were deposited at the Natural History Museum, London, United Kingdom (**NHMUK**) and at the Nakhon Phanom University, Faculty of Science, Thailand (**NPU**).

## Taxonomic section

### Order Calanoida Sars, 1903

#### Family Diaptomidae Baird, 1850

##### Genus *Mongolodiaptomus* Kiefer, 1938

###### Mongolodiaptomus loeiensis sp. n.

Taxon classificationAnimaliaCalanoidaDiaptomidae

http://zoobank.org/898BBF26-8F13-40B3-8A3F-EC36579FA3B1

**Figure 1.**
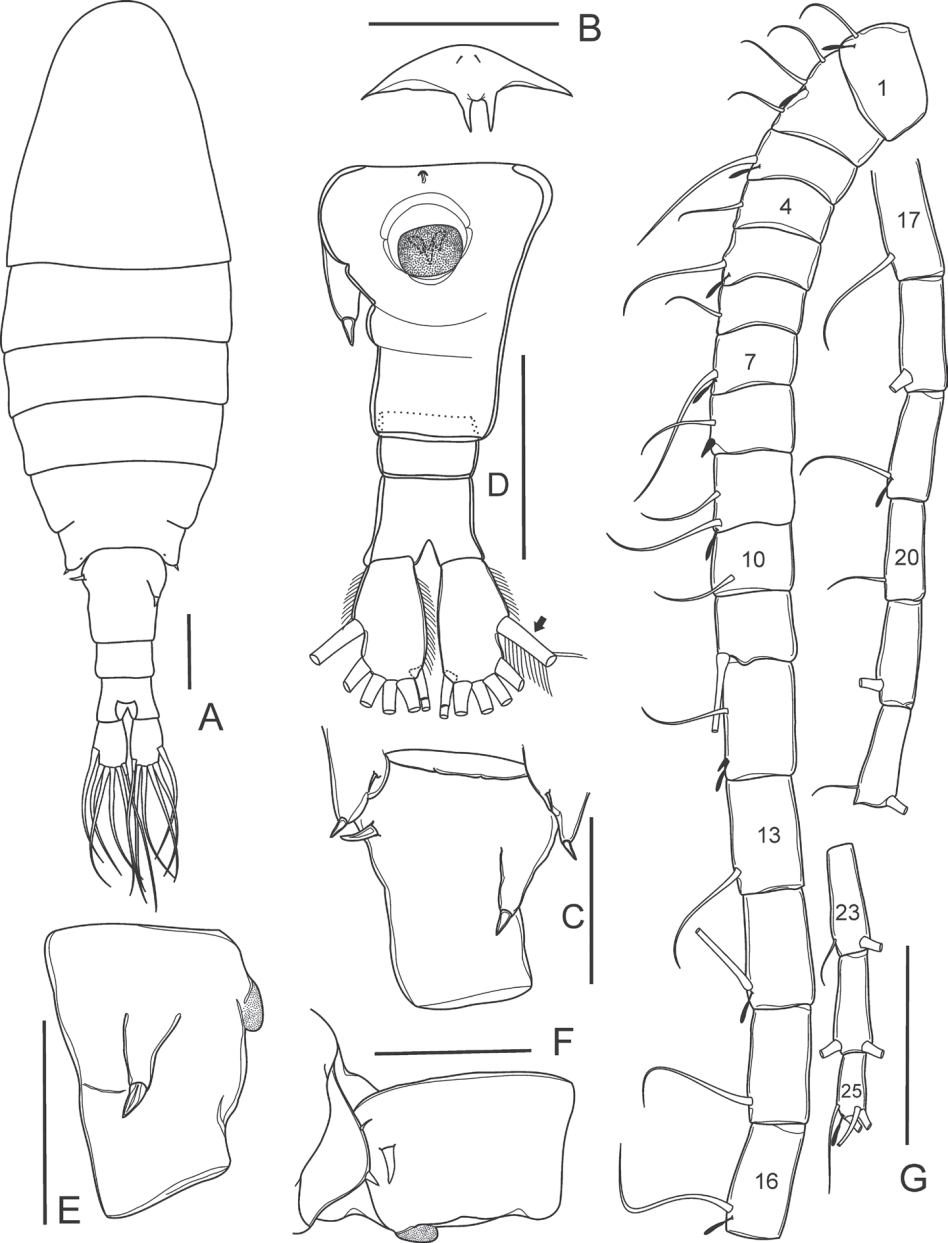
*Mongolodiaptomus loeiensis* sp. n. Female: **A** habitus, dorsal view **B** rostrum, frontal view **C** lateral wings on pediger 5 and genital somite, dorsal view **D** urosome, ventral view (black arrow points to smooth region of lateral seta) **E** genital somite, lateral view **F** lateral wing on pediger 5 and genital somite, lateral view **G** antennule. Scale bar 100 µm.

**Figure 2.**
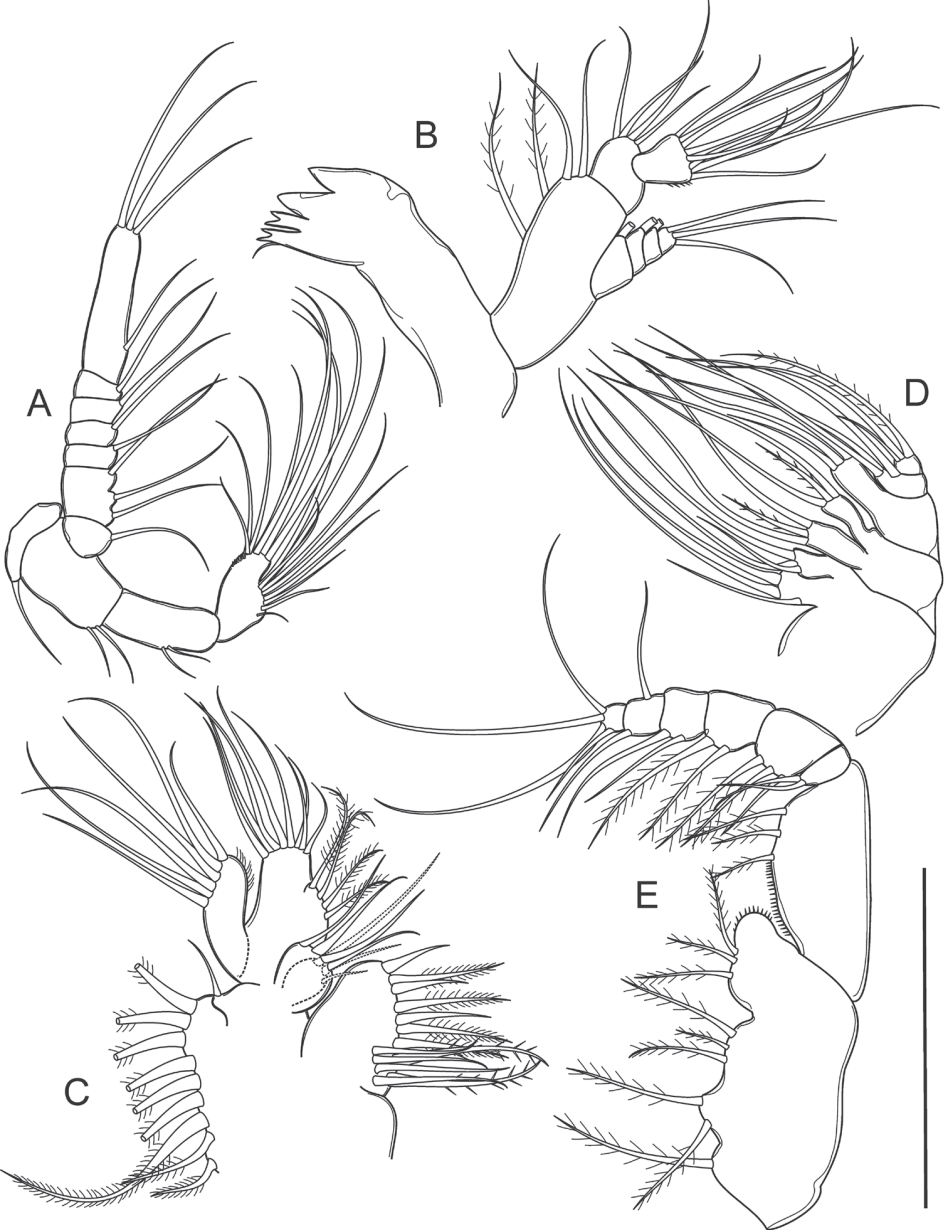
*Mongolodiaptomus loeiensis* sp. n. Female: **A** antenna **B** mandible **C** maxillule **D** maxilla **E** maxilliped. Scale bar 100 µm.

**Figure 3.**
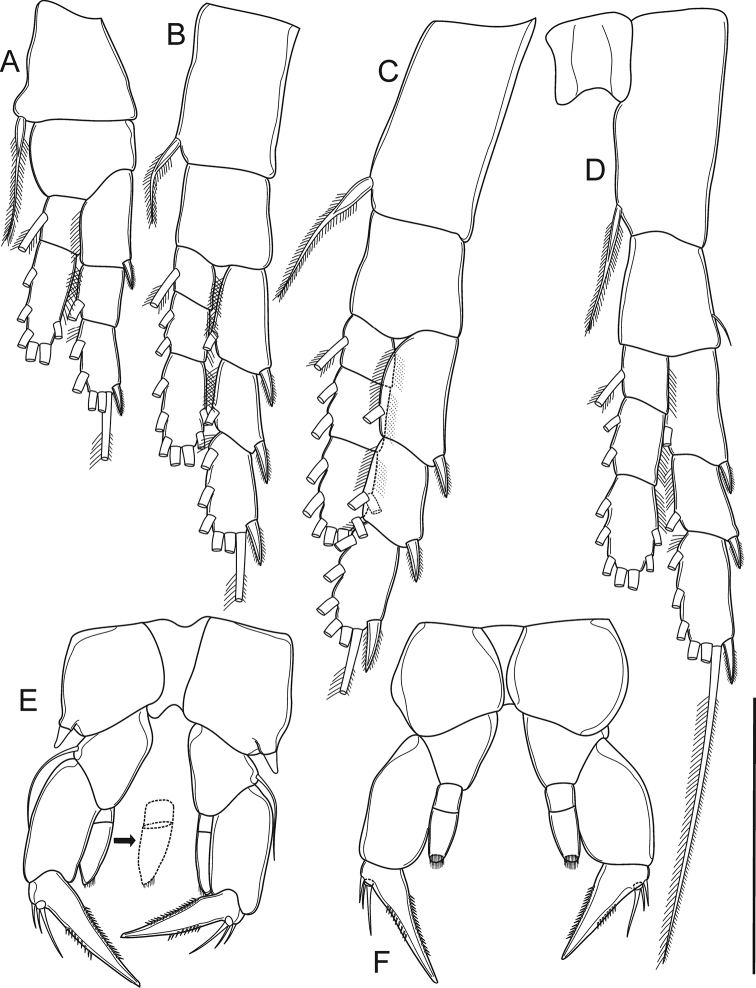
*Mongolodiaptomus loeiensis* sp. n. Female: **A** P1 **B** P2 **C** P3 **D** P4 **E** P5 in caudal view **F** P5 in frontal view. Scale bar 100 μm.

**Figure 4.**
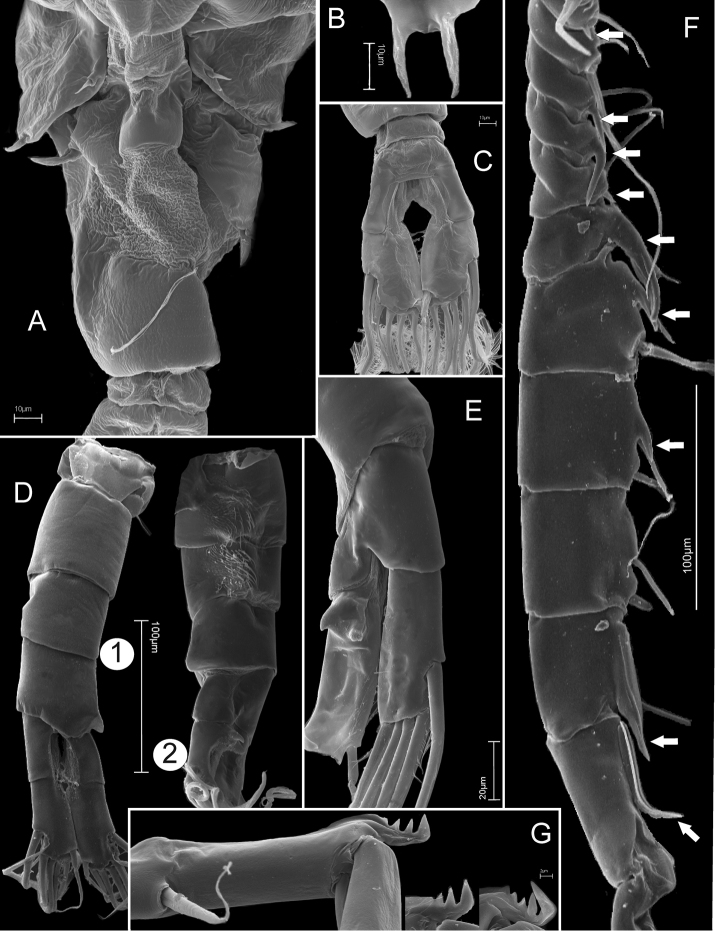
*Mongolodiaptomus loeiensis* sp. n., SEM photographs. Female (**A–C**): **A** pediger 5 and genital somite, dorsal view **B** rostrum, frontal view **C** urosomite 2, anal somite and caudal rami, dorsal view; Male (**D–G**): **D1** urosome in dorsal view **D2** urosome in ventro-lateral view **E** anal somite and caudal rami, ventral view **F** the right antennule segments 8–18 (white arrows point to spines) **G** spinous process on right antennule segment 20.

**Figure 5.**
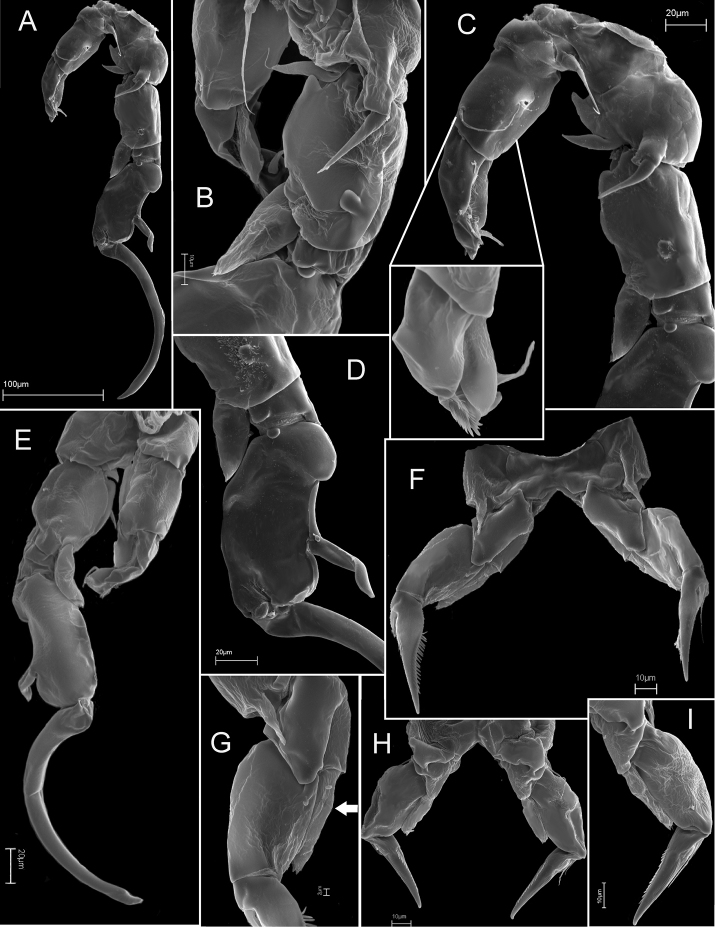
*Mongolodiaptomus loeiensis* sp. n., SEM photographs. Male (**A–E**): **A** P5 in caudal view **B** coxa, basis, Exp-1 of the right P5 in caudal view **C** intercoxal plate, coxa, basis, Enp of the right P5 and coxa, basis, Exp and Enp of the left P5, caudal view **D** Enp and Exp of the right P5 in caudal view **E** P5 in frontal view; Female (**F–I**): **F** P5 in caudal view **G** basis, Exp-1 and Enp in caudal view (white arrow indicated the segmented point of Enp) **H** P5 in frontal view **I** the left P5 in frontal view.

**Figure 6.**
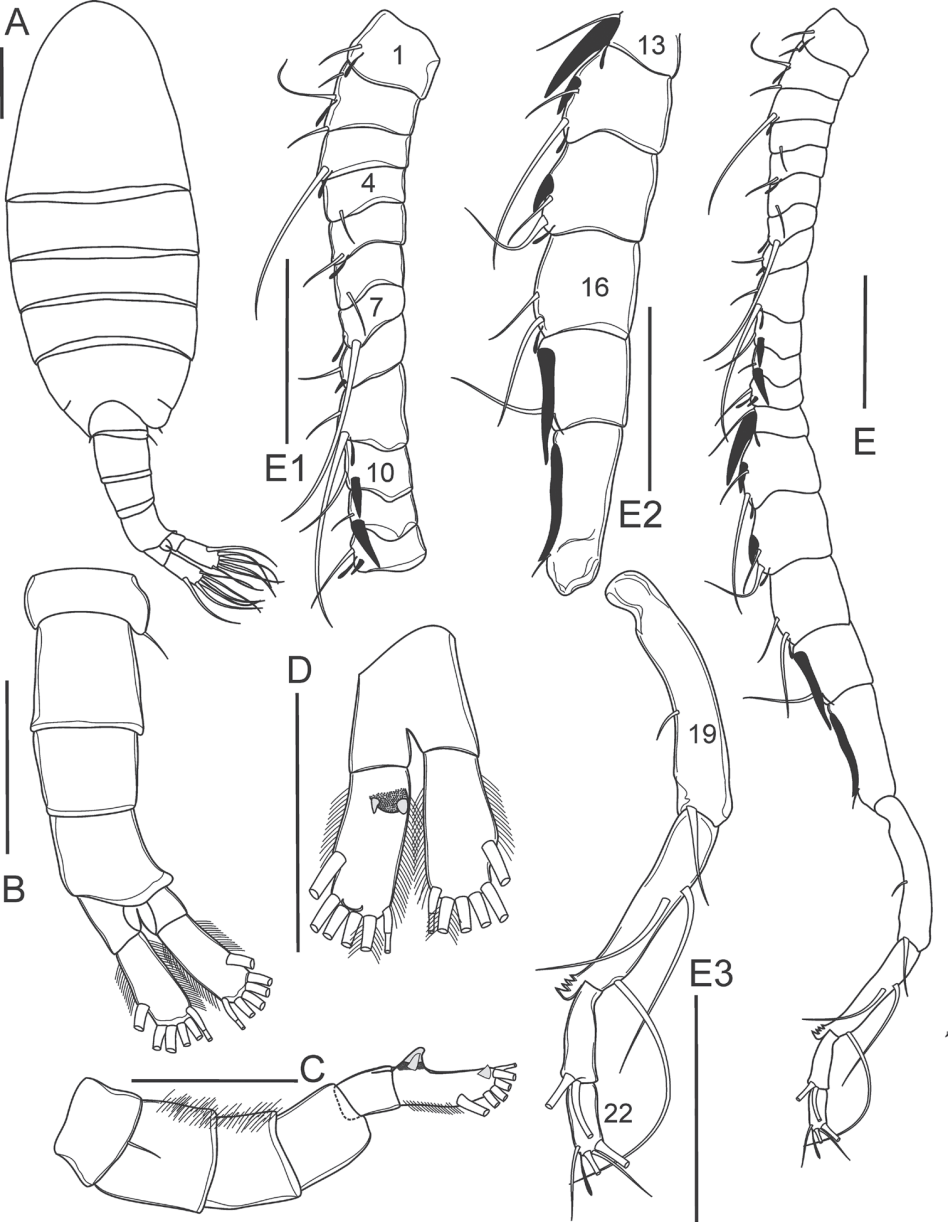
*Mongolodiaptomus loeiensis* sp. n. Male: **A** habitus, dorsal view **B** urosome, dorsal view **C** urosome, lateral view **D** anal somite and caudal rami, ventral view **E** antennule **E1** segments 1–12 **E2** segments 13–18 **E3** segments 19–22. Scale bar 100 µm.

**Figure 7.**
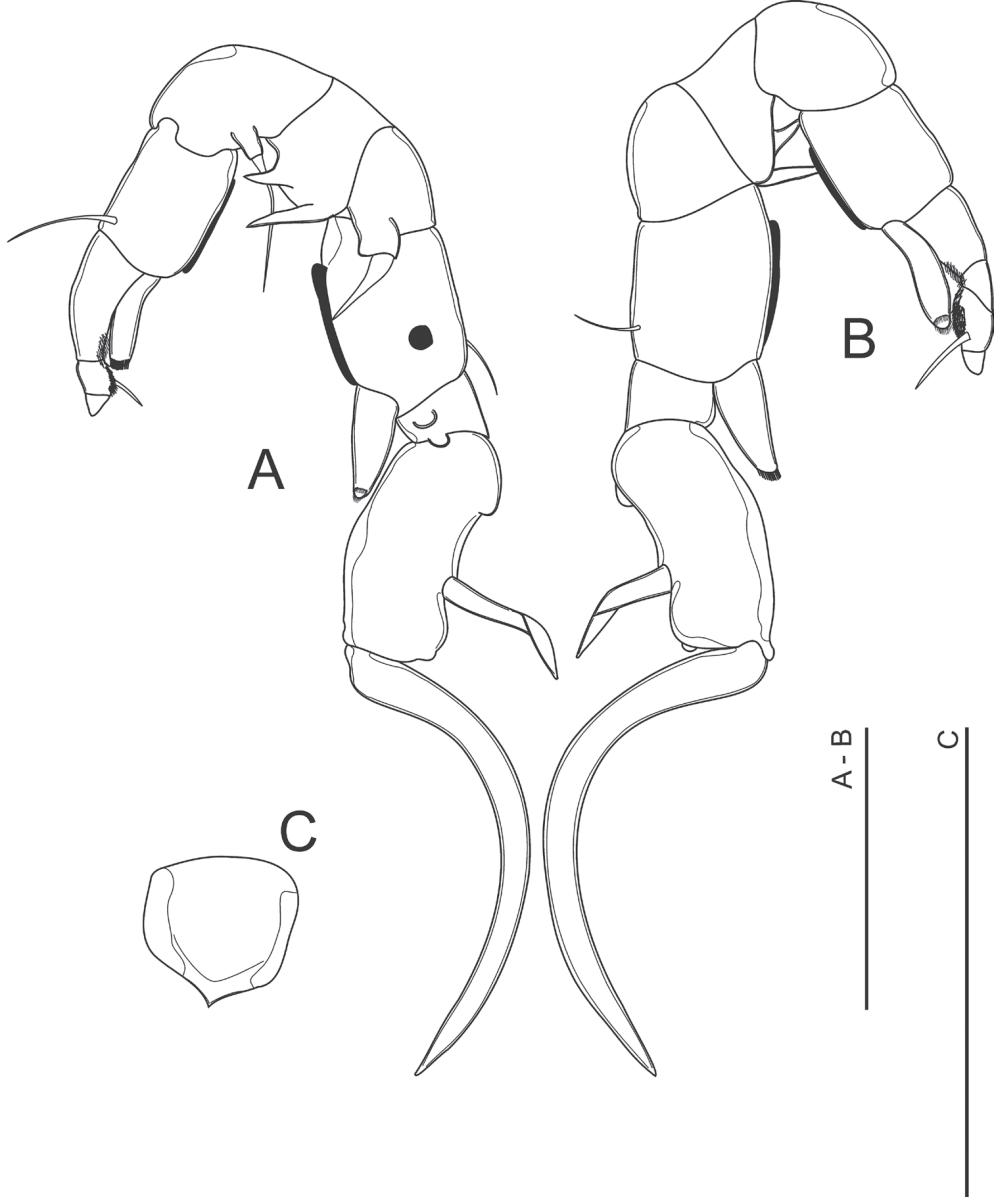
*Mongolodiaptomus loeiensis* sp. n. Male: **A** P5 in caudal view **B** P5 in frontal view **C** Right P5 Exp-1 in outer lateral view. Scale bar 100 μm.

Figs 1 , 2 , 3 , 4 , 5 , 6 , 7

####### Type locality.

A temporary pond nearby the Prakaipetch Cave, Nadokkham Subdistrict, Na Duang District, Loei Province, northeastern Thailand; 17°54'23"N, 101°54'23"E; altitude: 420 m above sea level.

####### Holotype.

One adult male, NHMUK 2017.134, dissected and mounted in glycerol on one slide: collected on 5 August 2015 by S. Watiroyram.

####### Allotype.

One adult female, NHMUK 2017.135, dissected and mounted in glycerol on one slide: collected on same date by the same collector.

####### Paratypes.

Ten adult females and males, NHMUK 2017.136–145, undissected and preserved in 70% ethanol in 1.5 ml microtube; 10 adult females and males, NPU 2017–001, undissected and preserved in 70% ethanol in 1.5 ml microtube: collected on same date by the same collector.

####### Differential diagnosis.

The right P5 Exp-2 of male in *Mongolodiaptomus loeiensis* sp. n. with principal spine slightly posterior to mid-outer margin and one spinous process each proximally and distally, fits the diagnostic features of the genus *Mongolodiaptomus* sensu Reddy et al. (2000).

The male of new species is most similar to *M. calcarus* by the segment 20 of right antennule with serrated spine, and the armature of P5: on right P5 (1) the presence of strong and stout coxal spine, (2) Exp-2 with similar shape and size, and (3) Exp-2 with strong and bent principal lateral spine; on left P5 (1) basis with long and narrow hyaline membrane on inner margin, and (2) Exp-2 with row of strong spinules on inner margin at distal half of segment. However, *M. loeiensis* sp. n. differs from *M. calcarus* by following morphological characters: (1) the new species with three chitinous processes on ventral surface of the right caudal ramus while *M. calcarus* with only two chitinous processes, (2) intercoxal plate of the new species with outgrowth process into two-spine like lobes at distal margin while *M. calcarus* without any outgrowth process, (3) basis of right P5 in the new species with inner hyaline membrane and with sub-globular chitinous process on caudal surface while *M. calcarus* without hyaline membrane, and with spur-like chitinous process, (4) the new species with long and slender end claw while it is typical short and robust in *M. calcarus*. Additional differences occur in female characteristics as follows: (1) the genital somite of *M. loeiensis* sp. n. with bulges on proximal, sub-middle, and middle region at right margin while *M. calcarus* is slightly convex on those region, (2) the right side of genital somite with spine located on outgrowth process in *M. loeiensis* sp. n. but it is directly inserted on somite in *M. calcarus*, (3) the left side of genital somite with slightly proximal dilated and spine inserted on small prominence while *M. calcarus* with obviously proximal rounded lobe and spine directly inserted on its segment.

####### Description of adult female.

Body length (Fig. 1A), measured from anterior margin of rostrum to posterior margin of caudal rami, 1.0–1.3 mm. Rostral spines (Figs 1B, 4B) with two teeth-like process in anterior margin. Prosome (Fig. 1A) ovoid, with cephalosome and 5 pedigers; pediger 4 and 5 fused, partly separated at lateral side. Pediger 5 (Figs 1C, 4A) with symmetrical postero-lateral wings, reaching proximal part of genital somite; each wing with one inner and one posterior spine on posterolateral margins (former spine smaller than later one). Urosome (Fig. 1A, D) consisting of 3-somites including genital somite, urosomite 2 and anal somite, approximately 1/2 as long as prosome. Genital somite (Figs 1C–F, 4A) exceeding in length urosomite 2, anal somite and caudal ramus combined. Right side: with dilated laterally in three regions on proximal half of segment length; with postero-laterally directed outgrowth (sub-conical process), extended beyond half of somite; with a short and blunt posterior spine on respective outgrowth. Left side: with regular margin, with a spine on dorso-lateral surface at proximal part, larger and sharper than the right spine. A pair of gonopores located beneath a genital operculum on mid-ventrally proximal part. Urosomite 2 symmetrical, shorter than latter somite. Anal somite (Figs 1A, D, 4C) slightly expanded at distal end, almost as long as caudal rami; anal operculum small and slightly concave on its posterior margin.

Caudal rami (Figs 1D, 4C) symmetrical, each ramus slightly expanded on distal end, about 1.6 times as long as wide; with a row of setules along inner and outer margins. Each ramus with six setae (seta II–VII): lateral (II) seta with smooth region on proximally outer margin; dorsal seta (VII) proximally jointed, bare, and longest.

Antennule (Fig. 1G) symmetrical, 25-segmented, reaching beyond the end of caudal setae. Setal formula starting from the first to the last segment (a = aesthetasc, s = spine): 1+a, 3+a, 1+a, 1, 1+a, 1, 1+a, 1+s, 2+a, 1, 1, 1+a+s, 1, 1+a, 1, 1+a, 1, 1, 1+a, 1, 1, 2, 2, 2, 4+a.

Antenna (Fig. 2A) 11-segmented. Coxa with one seta on distal corner. Basis with two inner setae on distal corner. Exp-1–6 with 1, 3, 1, 1, 1, and 1 inner seta, respectively. Exp-7 with one inner and three apical setae. Enp-1 with two inner setae. Enp-2 with nine inner and seven apical setae.

Mandible (Fig. 2B) with four strongly chitinized teeth and a single seta on gnathobase. Basis with four inner setae. Enp 2-segmented: Enp-1 with four inner setae, Enp-2 with nine apical setae plus tiny spinules along outer margin. Exp 4-segmented, with 1, 1, 1, and 3 setae, respectively.

Maxillule (Fig. 2C) with seven spines and six setae on praecoxal arthrite. Coxal endite and coxal epipodite with three and nine setae, respectively. Proximal and distal endites each with four setae; basal exite with one seta. Enp reduced, represented by eight apical setae. Exp with six setae plus a row of setules on median margin.

Maxilla (Fig. 2D) with three setae on proximal praecoxal and coxal endites, and distal praecoxal and coxal endites. Allobasis with three setae. Enp reduced to two segmented, each with three setae.

Maxilliped (Fig. 2E) with four endites on syncoxa: 1, 2, 3, 3 setae inserted on respective endites; endite 4 with tiny spinules on distal corner. Basis with three setae, ornamented with spinules on proximal half of segment. Enp 6-segmented, with 2, 3, 2, 2, 2, and 4 setae, respectively.

P1–P4 (Figs 3A–D), biramous, Exp longer than Enp. P1 with 3-segmented Exp and 2-segmented Enp, P2–P4 with 3-segmented Exp and Enp. Exp and Enp with longitudinal setules on inner and outer margin, respectively. Armature formula of P1–P4 as follows (legend: outer-inner seta/spine; outer-apical-inner; Arabic numerals represent setae, Roman numerals represent spines):

**Table:** 

	Coxa	Basis	Exopod			Endopod		
			1	2	3	1	2	3
P1	0-1	0-0	I-1	0-1	I-3-2	0-1	1-2-3	----
P2	0-1	0-0	I-1	I-1	I-3-3	0-1	0-2	2-2-3
P3	0-1	0-0	I-1	I-1	I-3-3	0-1	0-2	2-2-3
P4	0-1	1-0	I-1	I-1	I-3-3	0-1	0-2	2-2-3

P5 (Figs 3E–F, 5F–I) symmetrical. Coxa with a blunt, stout spine on protuberance at distal outer corner on caudal surface. Basis with a thin, bare seta on outer margin, reaching middle of Exp-1. Exp 3-segmented and Enp 2-segmented. Exp-1 sub-rectangular, more than 2.0 times as long as wide, slightly longer than Enp. Exp-2 sub-triangular, drawn out into claw-like, with a row of strong spinules along middle of both margins; with short and bare lateral spine. Exp-3 reduced into small prominence, with short lateral spine, and long apical seta. Enp subconical, Enp-1 rectangular, slightly shorter than wide. Enp-2 narrowed distally, with a circular row of spinules on distal end.

Additional ornamentation of P1–P5 as in Figs 3A–F, 5F–I.

Adult females with a single egg sac containing 8–10 eggs.

####### Description of adult male.

Body length (Fig. 6A) measured from anterior margin of rostrum to posterior margin of caudal rami, 0.9 –1.1 mm (mean = 1.0 mm, n = 5), smaller than female. Prosome as in female but lateral wings not well developed compared to those in female, pediger 5 without inner spine inserted on dorso-posterior margin of each wing. Urosome (Figs 4D, 6B–C) 5-segmented and asymmetrical, oriented downward to right side. Genital somite (Fig. 6B) dilated postero-laterally on right side accompanied with a small seta on distal corner. Urosomites 2–3 (Figs 4D, 6C) with a field of long hairs ventrally along middle of segment. Urosomite 4 (Figs 4D–E, 6B) with posterolateral dilated on right side; dorso-posterior margin expanded beyond anal operculum. Anal somite (Figs 4D–E, 6B–D) similar to female but asymmetrical, right side located at lower position than opposite side. Caudal rami (Figs 4D–E, 6B–D) similar to female in setation but having different shape and ornamentation on right ramus. Ramus asymmetrical and cylindrical shaped: right ramus with three ventral chitinous prominences: two teeth-like on large bulge at proximal region (outer one small and with sharped tip; inner one large and with rounded tip), and one semi-circular ridge located above insertion of caudal seta IV and V.

Antennule (Figs 4F–G, 6E) asymmetrical, reaching beyond end of caudal setae. Right antennule 22-segmented, setal formula (a = aesthetasc, s = spine): 1+a, 3+a, 1+a, 1, 1+a, 1, 1+a, 1+s, 2+a, 1+s, 1+s, 1+a+s, 1+a+s, 2+a+s, 2+a+s, 2+a, 2+s, 1+s, 2, 3+s, 2, 4+a; geniculated between segment 18 and 19; segment 20 (antepenultimate) with comb-like spine (3–5 teeth).

Left antennule, antenna, mouthparts, and P1–P4 similar to those in female.

P5 (Figs 5A–E, 7) asymmetrical, right leg reaching beyond caudal setae. Intercoxal plate with two tooth-like lobes on distal margin, its tip bent forward to left leg. Right P5: coxa with strong and stout spine inserted on well-developed posterior lobe on caudal surface. Basis with long and narrow hyaline lamella at inner margin; small chitinous prominence (sub-globular in shape) located approximately mid-distal of segment on caudal surface; distal outer margin with short, thin seta on frontal surface. Exp 2-segmented: Exp-1 small and shorter than wide, approximately 0.6 times as long as wide; with two prominent knobs on caudal surface; distal outer corner produced. Exp-2 enlarged, approximately 2.0 times as long as wide; proximal and distal part enlarged in similar size, inner margin slightly convex and outer margin concave; with two minute knobs and one principal (lateral) spine at outer margin (knob-like projection located on proximal and distal region; lateral spine inserted slightly anterior to mid-outer margin of segment). Lateral spine strong, cylindrical, approximately 1/2 of segment length; its tip bent upward to posterolateral direction on caudal view. End claw sickle-shaped, strong and pointed tip; approximately 1.5 times as long as Exp-2, inner and outer margins smooth. Enp 1-segmented, conical, reaching to proximal expansion of Exp-2, with cluster of spinules at rounded tip. Left P5: coxa with thin seta inserted on posterior lobe at distal inner corner, exceeding basis, and posterolateral margin with semi-circular concave on caudal surface. Basis with long, narrow hyaline lamella at inner margin (small size than right P5); with long, thin seta at posterolateral margin on caudal surface. Exp 2-segmented: Exp-1 longer than wide, gradually tapering posteriorly, inner margin concave and outer one convex; with field of setules on inner margin at distal end. Exp-2 smaller than Exp-1, conical; with seta on inner margin at distal end on frontal surface, as long as segment; with a cluster of strong spinules along inner margin; apical process stout, bare, and short. Enp 1-segmented, longer than Exp-1, with a cluster of spinules at its tip.

####### Etymology.

The specific name *loeiensis* refers to the place “Loei” where the new species was first recognized. The name with the Latin suffix “-*ensis*” is an adjective for place.

####### Distribution and ecology of the new species and other diaptomids in the area of study.

*Mongolodiaptomus loeiensis* sp. n. was found in a temporary pond with a mean water temperature of 25.5 °C, conductivity 259 μS/cm, and pH 7.6. The new species was a single calanoid copepod occurring in type locality. However, it was found co-occurring with other microcrustaceans, i.e. *Diaphanosoma excisum* Sars, 1885, *Thermocyclops decipiens* (Kiefer, 1929), and *Mesocyclops thermocyclopoides* Harada, 1931. In over 3,000 samples collected from Thailand (59 samples from Loei Province provided by the first author and about 3,000 samples from other provinces by the second author and her colleagues), the new species can by considered as a rare species because it was present only in the type locality from Loei Province (Sanoamuang 2002).

The occurrence of the new species is similar to that of *Phyllodiaptomus thailandicus* Sanoamuang & Teeramaethee, 2006 and Tropodiaptomus cf. ruttneri (Brehm, 1923), which have so far been known as rare species in Thailand and here each species was found in a single location or in about 2% of collected samples. *P. thailandicus* has hitherto been found in six provinces; Prachinburi, Chanthaburi, Sa Kaeo, Suphanburi, Kanchanaburi, and Chumphon (Sanoamuang and Teeramaethee 2006, Koompoot and Sanoamuang 2012) whereas Tropodiaptomus cf. ruttneri was only known from Phayao and Nan provinces (Sanoamuang 2002). The findings of *P. thailandicus* and Tropodiaptomus cf. ruttneri in this study are a new record to northeastern Thailand and provide more understanding on its distribution range in the country. *Vietodiaptomus blachei* (Brehm, 1951) was found in a few samples or approximately 9% of collected samples whereas *P. praedictus*, *M. botulifer*, and *M. calcarus* were frequently found in about 41, 39 and 6% of collected samples, respectively.

The ranges of water variables for four other diaptomids species collected from Loei Province areas are as follows: Tropodiaptomus cf. ruttneri in water with temperature 26.9–30.1 °C, conductivity 81–100 μS/cm, and pH 7.1–7.3; *P. thailandicus* = 25.0–30.1 °C, 310–360 μS/cm, and pH 7.3–7.5; *M. calcarus* = 23.6–31.2 °C, 197–287 μS/cm, and pH 7.6–8.0; *M. botulifer* = 26.0–30.7 °C, 57–343 μS/cm, and pH 7.2–7.8; *P. praedictus* = 24.6–31.8 °C, 57–386 μS/cm, and pH 7.6–8.2; *V. blachei* = 26.0–31.4 °C, 92–527 μS/cm, and pH 7.1–8.1.

## Discussion

The genus *Mongolodiaptomus* currently contains 12 species, *M. birulai*, *M. botulifer, M. calcarus*, *M. dumonti*, *M. formosanus*, *M. gladiolus*, *M. malaindosinensis*, *M. mephistopheles*, *M. pectinidactylus*, *M. rarus*, *M. uenoi*, and *M. loeiensis* sp. n. (Sanoamuang 2001, 2002 and the present study). Apart from the closest species *M. calcarus*, the male of the new species differs from its congeners by the following unique characters. The antepenultimate segment of right antennule has a comb-like spine in the new species versus it is smooth in *M. birulai*, *M. formosanus*, *M. botulifer* and *M. malaindosinensis*. The right caudal ramus has three ventral chitinous prominences in *M. loeiensis* sp. n. versus respectively 1, 1, 1, 1, none, 2 chitinous prominences (prominence is unknown for *M. gladiolus*) in *M. birulai*, *M. formosanus*, *M. mephistopheles*, *M. rarus*, *M. pectinidactylus*, and *M. dumonti*. Although right caudal ramus in *M. botulifer*, *M. malaindosinensis* and *M. uenoi* has three chitinous prominences, their shapes are different to that of the new species; *M. loeiensis* sp. n. has two teeth and one semi-circular ridge while *M. botulifer* and *M. malaindosinensis* have one tooth and two knobs, and *M. uenoi* has one tooth and two semi-circular ridges.

The male P5 of *M. loeiensis* sp. n. can be distinguished from its congeners by the following characters: (1) two spine-like lobes on distal margin of the intercoxal plate in the new species versus a triangular lobe in *M. birulai, M. botulifer, M. formosanus, M. pectinidactylus*, and *M. malaindosinensis* and one spine-like lobe in *M. uenoi* and no outgrowth in *M. dumonti*, *M. gladiolus, M. mephistopheles*, and *M. rarus*; on the right P5, (2) the coxal spine of the new species stronger and more robust compared to those in other species (except *M. uenoi*), (3) basis of the new species with a hyaline membrane on inner margin, which is absent in *M. gladiolus* and *M. dumonti*, (4) chitinous prominence present on caudal surface on the basis of the new species while absent in *M. birulai*, *M. formosanus*, *M. gladiolus*, *M. mephistopheles*, *M. malaindosinensis*, and *M. uenoi* (*M. botulifer* has chitinous ridge), (5) Exp-1 of the new species with produced process at distal outer corner versus absent in *M. birulai*, *M. dumonti*, *M. gladiolus*, *M. pectinidactylus* and *M. rarus*, (6) Exp-2 of the new species with a bent principal lateral spine versus straight in *M. birulai*, *M. gladiolus*, *M. dumonti*, *M. pectinidactylus* and *M. uenoi*, (7) on left P5 basis of the new species presence of a hyaline membrane on inner margin versus absent in *M. gladiolus* and *M. formosanus*. In the female, the genital somite of *M. loeiensis* sp. n. has a large posterolaterally directed outgrowth on the right side which is absent in the others (except *M. botulifer*, *M. gladiolus*, *M. malaindosinensis* and *M. rarus*).

### Key to worldwide species of *Mongolodiaptomus* Kiefer, 1938

**Table:** 

**Males**:
1	Spinous process on antepenultimate segment smooth	**2**
–	Spinous process on antepenultimate segment serrate	**5**
2	P5 Enp 1-segmented	**3**
–	P5 Enp 2-segmented	**4**
3	The basis of left P5 without hyaline membrane on inner margin	***M. formosanus***
–	The basis of left P5 with hyaline membrane on inner margin	***M. birulai***
4	The basis of right P5 with triangular hyaline membrane on inner margin	***M. malaindosinensis***
–	The basis of right P5 with spherical hyaline membrane on inner margin	***M. botulifer***
5	Intercoxal plate of P5 with outgrowth on distal margin	**6**
–	Intercoxal plate of P5 without outgrowth on distal margin	**8**
6	The principal lateral spine on Exp-2 of right P5 straight	**7**
–	The principal lateral spine on Exp-2 of right P5 bent	***M. loeiensis* sp. n.**
7	Intercoxal plate of P5 with rounded lobe on distal margin	***M. pectinidactylus***
–	Intercoxal plate of P5 with spine-like lobe on distal margin	***M. uenoi***
8	The basis of right P5 with hyaline membrane on inner margin	***M. mephistopheles***
–	The basis of right P5 without hyaline membrane on inner margin	**9**
9	The basis of left P5 with hyaline membrane on inner margin	**10**
–	The basis of left P5 without hyaline membrane on inner margin	**11**
10	The principal lateral spine on Exp-2 of right P5 straight	***M. dumonti***
–	The principal lateral spine on Exp-2 of right P5 bent	***M. calcarus***
11	The basis of right P5 with chitinous spur on caudal surface	***M. rarus***
–	The basis of right P5 without any process on caudal surface	***M. gladiolus***
**Females**:
1	P5 Enp 1-segmented	**2**
–	P5 Enp 2-segmented	**8**
2	Genital somite with postero-laterally directed outgrowth on right side	**3**
–	Genital somite without postero-laterally directed outgrowth on right side	**6**
3	The left spine inserted on lobe-process of genital somite	***M. gladiolus***
–	The left spine inserted directly on genital somite	**4**
4	Genital somite with posterolateral bulging	**5**
–	Genital somite without posterolateral bulging	***M. uenoi***
5	P5 with long Enp, reaching beyond distal end of Exp-1	***M. malaindosinensis***
–	P5 with short Enp, not reaching distal end of Exp-1 (2/3 of Exp length)	***M. botulifer***
6	P5 Exp-3 absent	***M. birulai***
–	P5 Exp-3 present	**7**
7	Genital somite with larger spine on left side compared to right side	***M. mephistopheles***
–	Genital somite with similar sized spine on left and right side	***M. formosanus***
8	Genital somite with postero-laterally directed outgrowth on right side	**9**
–	Genital somite without postero-laterally directed outgrowth on right side	**10**
9	Genital somite with hyaline membrane at mid-laterally on right side	***M. rarus***
–	Genital somite without hyaline membrane at mid-laterally on right side	***M. loeiensis* sp. n.**
10	The right spine inserted on small lobe of genital somite	***M. pectinidactylus***
–	The right spine inserted directly on genital somite	**11**
11	Genital somite with larger spine on left side compared to right side	***M. dumonti***
–	Genital somite with larger spine on right side compared to left side	***M. calcarus***

## Supplementary Material

XML Treatment for Mongolodiaptomus loeiensis
